# Numerical simulations of the hard X-ray pulse intensity distribution at the Linac Coherent Light Source

**DOI:** 10.1107/S1600577517007032

**Published:** 2017-06-15

**Authors:** Tom Pardini, Andrew Aquila, Sébastien Boutet, Daniele Cocco, Stefan P. Hau-Riege

**Affiliations:** a Lawrence Livermore National Laboratory, Livermore, CA 94550, USA; b SLAC National Accelerator Laboratory, Menlo Park, CA 94025, USA

**Keywords:** XFEL, LCLS, wavefront propagation simulations, X-ray optics

## Abstract

Numerical simulations of the current and future pulse intensity distributions at selected locations along the Far Experimental Hall, the hard X-ray section of the Linac Coherent Light Source (LCLS), are provided. Estimates are given for the pulse fluence, energy and size in and out of focus, taking into account effects due to the experimentally measured divergence of the X-ray beam, and measured figure errors of all X-ray optics in the beam path. Out-of-focus results are validated by comparison with experimental data. Previous work is expanded on, providing quantitatively correct predictions of the pulse intensity distribution. Numerical estimates in focus are particularly important given that the latter cannot be measured with direct imaging techniques due to detector damage. Finally, novel numerical estimates of improvements to the pulse intensity distribution expected as part of the on-going upgrade of the LCLS X-ray transport system are provided. We suggest how the new generation of X-ray optics to be installed would outperform the old one, satisfying the tight requirements imposed by X-ray free-electron laser facilities.

## Abstract 2

Numerical simulations of the current and future pulse intensity distributions at selected locations along the hard X-ray section of the Linac Coherent Light Source are provided. Estimates are given for the pulse fluence, energy and size in and out of focus, taking into account effects due to the experimentally measured divergences of the X-ray beam, and measured figure errors of all X-ray optics in the beam path.

## 1. Introduction   

The Linac Coherent Light Source (LCLS) (Emma *et al.*, 2010 ▸) has been operational since 2009 as the first hard X-ray free-electron laser. The intense and femtosecond nature of its X-ray pulses has enabled the study of extremely weak and fast phenomena, ranging from the scattering from sub-micrometer crystals (Chapman *et al.*, 2011 ▸) to the fast demagnetization in solids following an intense perturbation (Bergeard *et al.*, 2015 ▸). Upgrades to the machine are constantly matched by increasingly complex user-driven experimental setups, whose success relies on estimates of the LCLS electric field distribution at the interaction plane. Computing the latter is rarely an easy task. For instance, the mirror-based transport system responsible for delivering the beam from the undulator exit to any experimental chamber can significantly affect the beam intensity distribution. The finite figure error of the nearly decade old LCLS transport mirrors (Soufli *et al.*, 2009 ▸; McCarville *et al.*, 2008 ▸; Barty *et al.*, 2009 ▸) produces aberrations resulting in diminished intensity at focus. Moreover, the actual performance of the lasing process may differ somewhat from the design parameters. This is the case for LCLS (Turner *et al.*, 2016 ▸), where a larger-than-expected beam divergence causes beam clipping off the edges of the transport mirrors originally designed for a smaller beam footprint. This effect was not known at the time of Barty *et al.*’s (2009 ▸) work, which appeared in the literature before LCLS came on-line. In this paper, we intend to provide the community with a current reference for the beam intensity distribution in and out of focus at the hard X-ray Far Experimental Hall (FEH) of LCLS. We provide results at three separate locations along the beam path: the entrance aperture of the FEH, and the 1 µm and the 0.1 µm focal planes of the CXI instrument (Boutet & Williams, 2010 ▸). Using wavefront propagation algorithms we show that the combination of beam divergence and imperfect X-ray transport mirrors causes aberrations out of focus that are larger than those previously estimated (Barty *et al.*, 2009 ▸). The same simulations show that this translates into a severe loss of integrated and peak intensity at the focus, where we obtain values for the beam fluence, full width at half-maximum (FWHM) and pulse energy. We believe the choice of FWHM as a metric for the quality of the intensity profile is justified by the small deviations from the ideal Gaussian profile observed in this work. These parameters are compared for reference with the same parameters one would obtain in the ideal scenario of infinitely long and perfectly figured X-ray mirrors. Given that LCLS is currently undergoing an upgrade of its X-ray transport mirrors, we conclude this work by providing quantitative estimates of the improvements that are expected. We do so including mirror figure specifications in our wavefront propagation simulations consistent with preliminary measurements obtained on these new substrates. The latter have shown remarkably small deviations from the ideal flat figure.

## 2. The FEH and the CXI instrument   

The FEH entrance aperture is located approximately 340 m downstream of the undulator exit. In order to reach it, the LCLS beam undergoes two reflections off two X-ray mirrors (hard X-ray offset mirror system; HOMS) (Soufli *et al.*, 2009 ▸), located approximately 70 m and 80 m, respectively, from the undulator exit. These mirrors were developed nearly ten years ago to state-of-the-art specifications at the time. The length of these mirrors measures 450 mm, and their clear aperture was intended to reflect the full footprint of the X-ray beam as given by original simulations of the undulator performance. Of the four mirrors that were polished, #4 and #2 have been installed at LCLS as the upstream and downstream mirrors, respectively. Their figure error measured with a Zygo 12-inch interferometer amounts to a remarkable 1.5 nm (#4) and 1.0 nm r.m.s. (#2) across the full aperture (Barty *et al.*, 2009 ▸). Such figure errors result in minor phase aberrations that propagate to amplitude errors out of focus (Zhou & Burge, 2010 ▸) with a corresponding loss of peak intensity in focus. The effect of these aberrations has already been captured by the numerical work of Barty and co-workers (Barty *et al.*, 2009 ▸). However, an important aspect of the LCLS performance was unknown at the time that work was published. Today it is clear that the divergence of the LCLS beam is approximately two times larger than anticipated (Turner *et al.*, 2016 ▸), with a corresponding beam waist that is smaller than predicted. As a result, the transport mirrors are undersized, and the footprint of the beam exceeds the mirrors’ clear aperture, extending past their physical size. As we will show later, this results in large phase aberrations at the edges coupled with beam clipping, leading to a (wavelength-dependent) loss in beam intensity at focus.

For the purpose of our simulations the CXI Instrument is located 380 m downstream of the undulator exit. The instrument is equipped with two experimental chambers featuring a 1 µm and 0.1 µm focal spot. This is achieved *via* two separate Kirkpatrick–Baez (KB) focusing systems featuring 370 mm-long substrates working at a grazing angle of 3.35 mrad, with a 350 mm-long clear aperture. The focal lengths of the KB systems (measured at the mid-point between the horizontal and vertical focusing mirrors) are 8.5 m and 0.7 m for the 1 µm and 0.1 µm systems, respectively.

## 3. The *XFELsim* wavefront propagation   

In order to quantify the intensity distribution of the LCLS hard X-ray beam, we perform wavefront propagation simulations using the Lawrence Livermore National Laboratory (LLNL) developed *XFELsim* end-to-end simulation capability of XFEL experiments. This package includes: (i) routines for the generation of self-amplified spontaneous emission (SASE) radiation fields *via* the *GENESIS* code (Reicher, 1999 ▸), (ii) wavefront propagation routines *via* the PROPER library of functions (Krist, 2007 ▸) translated into Python, and (iii) a selected number of routines for wavefront–sample interactions. Good agreement between the output of this package and the analytical model of Church & Tacaks (1995 ▸) has been shown in published work (Pardini *et al.*, 2015 ▸). Our simulations are two-dimensional in that the electric field is mapped along both axes perpendicular to the direction of the propagation. For the present study the spectral content of a SASE pulse is irrelevant, given that the energy-integrated intensity profile is investigated. Therefore, instead of using *GENESIS*, the beam is simply modeled as a Gaussian beam with an electric field distribution of the form
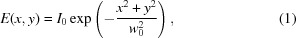
where 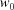 is the beam waist. The dependence of the latter on the experimentally measured FWHM divergence 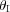 of the LCLS pulse *intensity* distribution (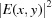) can easily be derived as
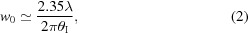
remembering that, according to Gaussian propagation, the *field* distribution half divergence 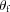 ≃ 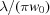 and 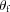 ≃ 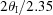. We set the photon energy to 8.0 keV, a value at which the experimentally measured 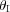 ≃ 4.75 µrad, leading to a value for the beam waist of 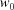 = 11.8 µm. X-ray mirrors are treated as finite-size apertures, and their as-measured height error is included where available. For the two FEH X-ray transport mirrors, the LLNL-measured figure error is included in the simulations (Barty *et al.*, 2009 ▸). The figure error of both 1 µm KB substrates is obtained from the work of Siewert and co-workers (Siewert *et al.*, 2012 ▸). Due to the lack of published data on the figure error of the 0.1 µm KB substrates, the latter has been randomly generated with the same r.m.s. height error as its 1 µm counterpart. The finite grid size of our numerical simulations limits the highest height error frequency accounted for to ∼0.25 mm^−1^. One-dimensional simulations employing larger grid sizes, or analytical models such as the one by Church & Takacs (1995 ▸), can be implemented to account for higher-frequency contributions to the intensity profile. The pulse energy is set to 1 mJ and is defined as
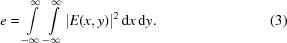
Given that LCLS routinely operates at pulse energies ranging from 1 to 6 mJ, our results can easily be scaled to any particular value of interest. We compute intensity distribution maps at the entrance aperture of the FEH, and at the 1 µm and 0.1 µm focal planes of the CXI instrument. The results are normalized to provide the correct pulse energy once the spatially dependent intensity is integrated over the pulse size.

## 4. Results   

**Figure 1:**
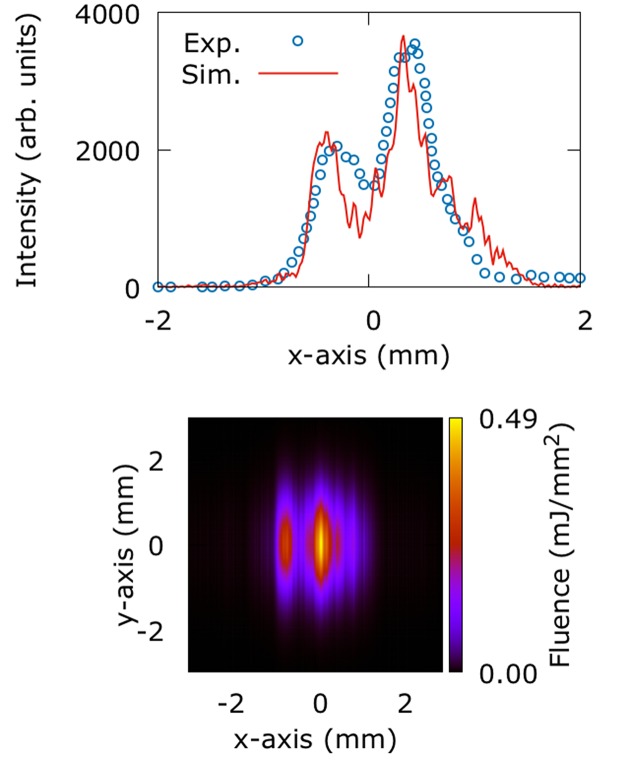
(Top) The experimentally measured (Turner *et al.*, 2016 ▸) and simulated intensity profile along the *x*-axis at the entrance aperture of the FEH for the current status of the machine. The simulated profile is obtained by integrating the two-dimensional distribution (bottom) along the *y*-axis. The *x*-axis is parallel to the optical axis of the transport mirror. The height error of the latter results in amplitude aberrations at the FEH. (Bottom) The two-dimensional simulated intensity distribution at the entrance aperture of the FEH for the current status of the machine. The *y*-axis intensity retains its almost ideal Gaussian profile.

The results of our simulations, including numerical values for the fluence, FWHM and pulse energy at selected locations along the FEH and the CXI instrument, are given in Figs. 1 ▸ ▸ ▸ ▸ to 5 ▸ and Tables 1 ▸ ▸ to 3 ▸. Also shown are simulated intensity distribution maps, where the maximum of the color bar matches the peak fluence in each case. In the following section we discuss our findings.

## 5. Discussion   

The first step in our simulations is the modeling of the beam intensity profile at the entrance aperture of the FEH. The motivation is twofold: on one hand the beam must be modeled at the entrance aperture in order to be propagated to any downstream location. On the other hand the beam intensity has been measured experimentally at this location, which offers us the chance to validate our results. Fig. 1 ▸ demonstrates that our results are in good agreement with the measurements, showing a beam that is visibly aberrated along the *x*-axis. The latter runs parallel to the X-ray transport mirror’s optical axis, along which the wavefront picks up phase aberrations eventually propagating into amplitude errors. The weight of the pulse is distributed among two intense lobes followed by a weaker tail towards the positive *x*-axis. This is mainly the result of the combined figure error of the two transport mirrors, while their finite length is responsible for the reduction to 0.88 mJ of the pulse energy. The large deviation from the ideal Gaussian profile makes the definition of the intensity FWHM difficult. However, an upper limit of 1.8 mm seems reasonable. We point out that the grouping of integrated intensity in two separate lobes yields a peak fluence (0.49 mJ mm^−2^) that is actually higher than that obtained in the ideal case (0.29 mJ mm^−2^), discussed later in this section, as shown in Table 1 ▸. As expected, given the lack of phase error imparted to the wavefront in the *y*-axis, the intensity profile along the latter is almost perfectly Gaussian.

We then propagate the beam separately to the 1 µm and 0.1 µm focal plane of the CXI instrument. No experimental data on the intensity distribution at focus is available today from direct imaging techniques. However, our simulations indicate that at the focal planes the pulse intensity distribution regains diffraction-limited behavior. For both 1 µm and 0.1 µm systems the numerically computed FWHM reported in Table 3 ▸ is consistent with diffraction off the edges of the 370 mm-long KB substrates at a grazing angle of 3.35 mrad. However, the larger-than-expected divergence of the LCLS beam combined with the finite size of the KB substrates causes a severe reduction of the pulse energy at focus down to 0.34 mJ. The corresponding fluence measures 0.2 mJ µm^−2^ and 18.1 mJ µm^−2^ for the 1 µm and 0.1 µm focal planes, respectively.

**Figure 3:**
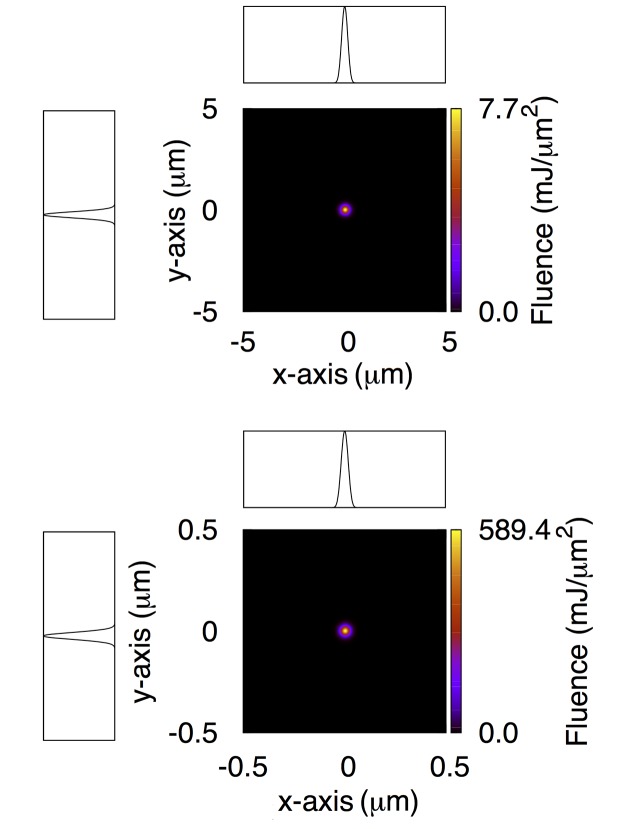
Intensity distribution at the 1 µm (top) and 0.1 µm (bottom) focal plane of the CXI instrument for the ideal scenario of perfectly figured and infinitely long mirrors. Lineouts are also shown. Each lineout has been obtained by integrating the two-dimensional intensity distribution along one of the axes. Spatial axis labels for the lineouts have been omitted for simplicity, and are the same as the corresponding two-dimensional maps.

In Fig. 3 ▸ we show the intensity distribution one would obtain at the focal planes in the ideal case of infinitely long and perfectly figured mirrors. We omit the intensity map at the entrance aperture of the FEH which obviously shows a perfectly Gaussian beam along both the *x*- and *y*-axes. The values of focal fluence, pulse energy and FWHM listed in Tables 1 ▸, 2 ▸ and 3 ▸ provide an absolute upper limit for the beam quality along this instrument.

**Figure 4:**
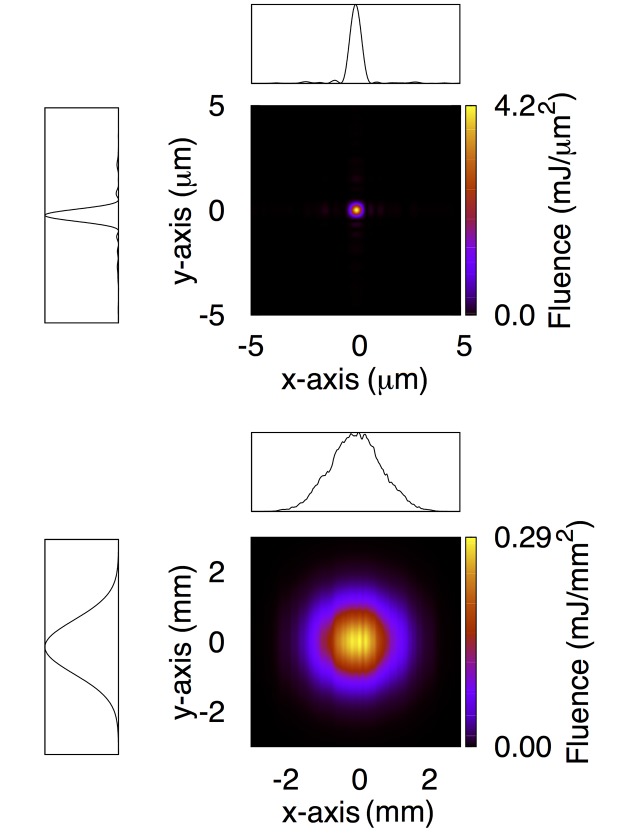
Estimated intensity distribution at the 1 µm focal plane (top) and at the entrance aperture of the FEH (bottom) after completion of the X-ray mirror upgrade. Lineouts are also shown. Each lineout has been obtained by integrating the two-dimensional intensity distribution along one of the axes. Spatial axis labels for the lineouts have been omitted for simplicity, and are the same as the corresponding two-dimensional maps.

**Figure 5:**
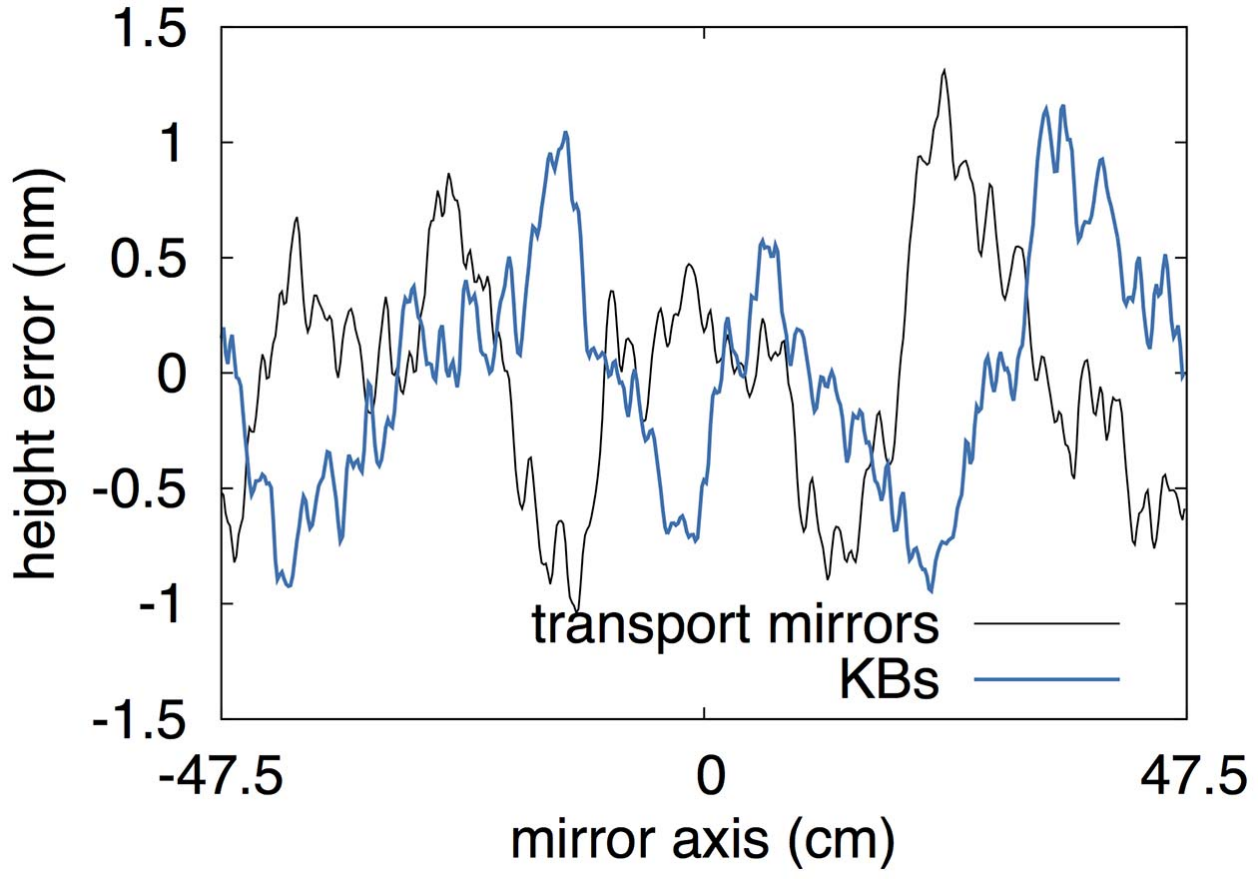
Randomly generated figure error for the new transport mirrors and the upgraded KB mirrors. See text for details.

The results from the ideal scenario are particularly important in order to appreciate the leap forward that may be enabled once the on-going upgrade of the LCLS hard X-ray transport mirrors is completed. As part of this upgrade, five new mirrors have been manufactured and delivered. Two of these mirrors will be installed in early 2017, replacing the current transport mirrors delivering X-rays to the CXI instrument. The improvements made to substrate polishing techniques over the last decade has yielded 950 mm-long substrates with a figure error measuring better than 0.5 nm r.m.s. over their entire length, and better than 0.3 nm in the central 300 mm. This is according to preliminary data available at the time this manuscript was prepared. We point out that the nearly 1 m length of the substrates removes the current clipping of the beam at lower energies, already improving beam quality. In order to quantify the improvements to the pulse intensity distribution in and out of focus, we repeat wavefront propagation simulations through the FEH down to the CXI instrument, with these new transport mirrors. Their height error profile is randomly generated over the full length to have a 0.5 nm r.m.s. figure error, and is then filtered through a measured height profile of a real X-ray optic to replicate the fractal form of the power spectral density typical of highly polished surfaces. The results are shown in Fig. 5 ▸. We consider the case where the focusing optics of the CXI instrument would also be replaced by mirrors with the same size and figure specifications. Given that the deployment of 950 mm mirrors within a 1 m focal length is unfeasible, we limit these calculations to the study of the 1 µm focal plane. Fig. 5 ▸ also shows the height error applied to the improved KB mirrors. Then our simulations show that the full 1 mJ pulse energy is transported to the entrance aperture of the FEH, with an ideal pulse FWHM and negligible aberrations, as shown in Fig. 4 ▸. This leads to a focused beam at the CXI 1 µm focal plane approaching 4.2 mJ µm^−2^ of fluence (54% of the ideal fluence), with a sub-micrometer FWHM along both axes. The latter is due to the large demagnification of the optical design, and the smaller-than-anticipated beam waist at the source.

## 6. Conclusions   

We have used our *XFELsim* simulation capability to perform wavefront propagation simulations of the beam intensity distribution at the FEH, the hard X-ray branch of LCLS. By taking into account the correct divergence of the LCLS hard X-ray beam and the specifications of all the X-ray mirrors in the beam path we computed realistic and current values for the pulse fluence, energy and size at three locations along the beam path: the entrance aperture of the FEH, and the 1 µm and 0.1 µm focal planes of the CXI instrument. We show how the height error of the current transport mirrors causes aberrations out of focus. These, combined with a larger-than-expected beam divergence, translate into a loss of fluence at both focal planes. We compare our results with the values one would obtain in the ideal scenario of infinitely long and perfectly figured optics. More importantly we provide estimates for the beam intensity distribution that is expected once an overall upgrade of the beam transport system is completed. We hope this work will provide a reference for users in the design, execution or data analysis phase of their experiments.

## Figures and Tables

**Figure 2:**
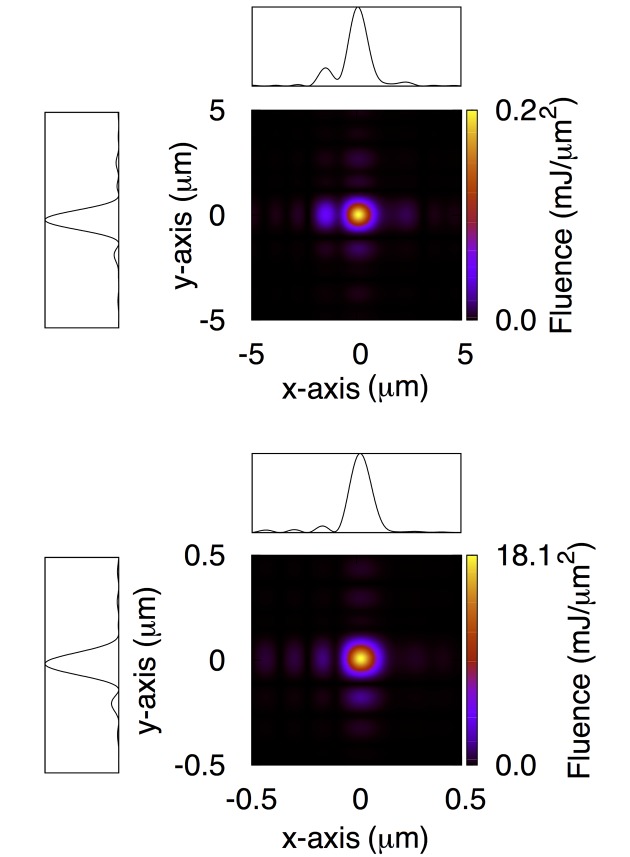
Intensity distribution at the 1 µm (top) and 0.1 µm (bottom) focal plane of the CXI instrument for the current status of the machine. Lineouts are also shown. Each lineout has been obtained by integrating the two-dimensional intensity distribution along one of the axes. Spatial axis labels for the lineouts have been omitted for simplicity, and are the same as the corresponding two-dimensional maps.

**Table 1:** Numerically computed peak fluence at selected locations along the beam path for (i) the current LCLS status, (ii) after completion of an overall upgrade of the LCLS mirrors, and (iii) for the ideal case of perfectly figured and infinitely long mirrors The current transport mirrors are responsible for binning the integrated pulse intensity in two separate lobes yielding a peak fluence (0.49 mJ mm^−2^) that is actually higher than that one would obtain in the ideal scenario of perfect mirrors (0.29 mJ mm^−2^). For the ideal case we also provide (in parentheses) the peak fluence one would obtain with perfectly figured finite-length mirrors (950 mm long). The finite size of the KB optics would be mainly responsible for the loss of peak fluence at focus.

	Peak fluence		
Beam location	Current	After upgrade (estimated)	Ideal
FEH entrance aperture (mJ mm^−2^)	0.49	0.29	0.29 (0.29)
1 µm focal plane (mJ µm^−2^)	0.20	4.20	7.70 (4.4)
0.1 µm focal plane (mJ µm^−2^)	18.1	–	589.4 (331.3)

**Table 2:** Numerically computed pulse energy at selected locations along the beam path for (i) the current LCLS status, (ii) after completion of an overall upgrade of the LCLS mirrors, and (iii) for the ideal case of perfectly figured and infinitely long mirrors

	Pulse energy (mJ)		
Beam location	Current	After upgrade (estimated)	Ideal
FEH entrance aperture	0.88	1.0	1.0
1 µm focal plane	0.34	0.93	1.0
0.1 µm focal plane	0.34	–	1.0

**Table 3:** Numerically computed FWHM of the intensity distribution at selected locations along the beam path for (i) the current LCLS status, (ii) after completion of an overall upgrade of the LCLS mirrors, and (iii) for the ideal case of perfectly figured and infinitely long mirrors The axis corresponding to each value is shown in parentheses.

	FWHM		
Beam location	Current	After upgrade (estimated)	Ideal
FEH entrance aperture (mm)	–	1.76 (*x*, *y*)	1.76 (*x*, *y*)
1 µm focal plane (µm)	1.05 (*x*), 0.94 (*y*)	0.43 (*x*, *y*)	0.34 (*x*, *y*)
0.1 µm focal plane (µm)	0.12 (*x*), 0.11 (*y*)	–	0.04 (*x*, *y*)
